# Operative managements of extracranial carotid artery aneurysms: a report of three cases and literature review

**DOI:** 10.1186/s41016-018-0143-6

**Published:** 2018-12-06

**Authors:** Maimaitituersun Abudula, Aximujiang Axier, Kaheerman Kadeer, Xiaojiang Cheng, Taotao Dou, Atawula Tuersun, Mierzati Tuerhong, Maimaitili Aisha

**Affiliations:** Department of Neurosurgery, The First Affiliated Hospital of Xinjiang Medical University, 137 Liyushannan Road, Urumqi, 830054 Xinjiang China

**Keywords:** Extracranial carotid artery aneurysm, Endovascular treatment, Surgical treatment, Willis covered stent

## Abstract

**Background:**

The purpose of this study is to report the treatment approaches and postoperative outcomes of extracranial carotid artery aneurysms (ECAAs) and discuss the symptoms, related risk factors, etiology, diagnostic methods, treatments, and follow-up period complications.

**Case presentation:**

We describe three patients with symptomatic extracranial carotid artery aneurysms; one of them was treated with end-to-end anastomosis of the extracranial internal carotid artery (EICA) after the resection of the aneurysm, while the other two patients were deployed with Willis covered stents in the extracranial internal carotid artery. All of the patients were in good condition when discharged home. After a mean follow-up period of 8 months, all the patients were alive and only one of them had the neurologic deficit. Additionally, we reviewed the relative literatures.

**Conclusion:**

Both of the surgical and endovascular treatments have relatively satisfactory outcomes in ECAA patients. However, it is necessary to provide individualized treatments to different patients according to the characteristics of the aneurysms.

## Background

Extracranial carotid artery aneurysms (ECAAs) are infrequent pathologies with an incidence of 0.4–4% of all aneurysms and pose a high risk of neurological thromboembolic events, cranial nerve compression, and more rarely rupture [1, 2]. Occurrence of the extracranial internal carotid artery (EICA) aneurysm is even more uncommon. The outcomes of large series and postoperative long-term follow-ups of ECAA have not been obtained yet. There is no consensus on how to treat ECAAs as they are rarely identified and operated. However, the most commonly utilized treatment methods are microsurgery and endovascular procedures. Therefore, the treatment methods are determined by the basic characteristics of the ECAAs as they vary and compose different challenges for neurosurgeons.

## Case presentation

### Case 1

**Fig. 1:**
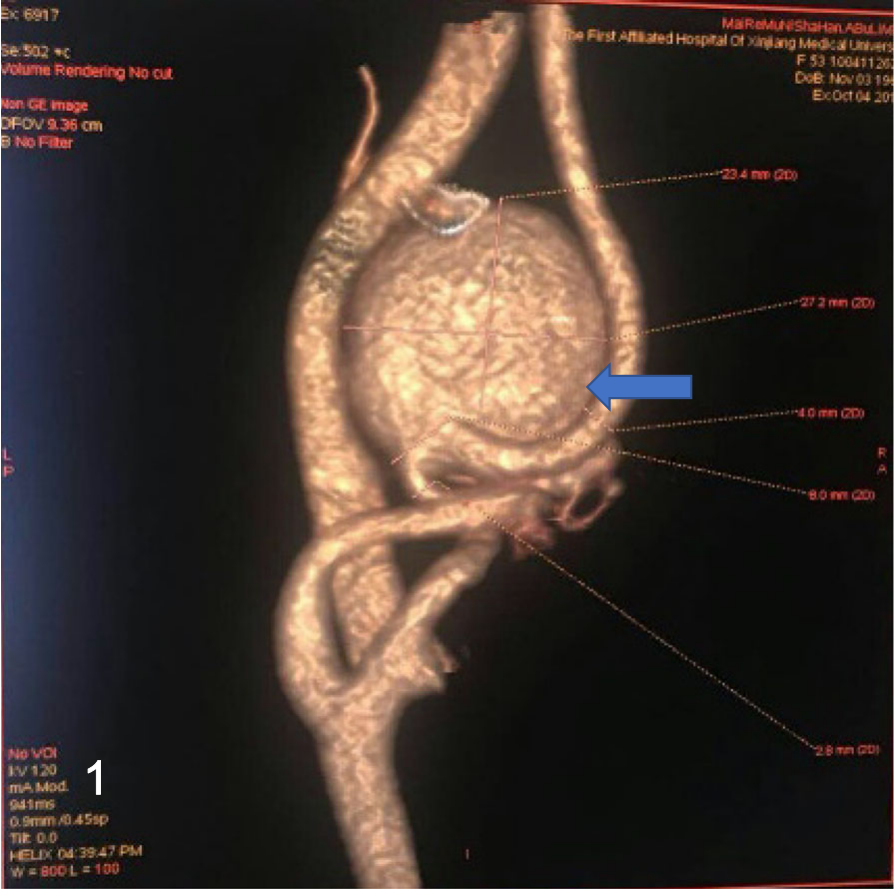
CTA showed the left EICA giant aneurysm (arrow), measuring the lesion was 27 × 23 mm in diameter

A 59-year-old female complained of finding a pulsatile mass on the left side of her neck for 5 years. During the recent 6 months, she had a slight headache with dizziness. Most importantly, the size of the mass increased rapidly, with its pulsatile frequency that was more evident than before. She presented no cervical infection, trauma, or surgery, but had a history of hypercholesterolemia and coronary artery disease for 9 years. On physical examination, we observed a pulsatile mass on her left neck with a palpable thrill and systolic bruit. Three-dimensional computed tomography angiography (CTA: Fig. 1) confirmed the giant aneurysm.

**Fig. 2:**
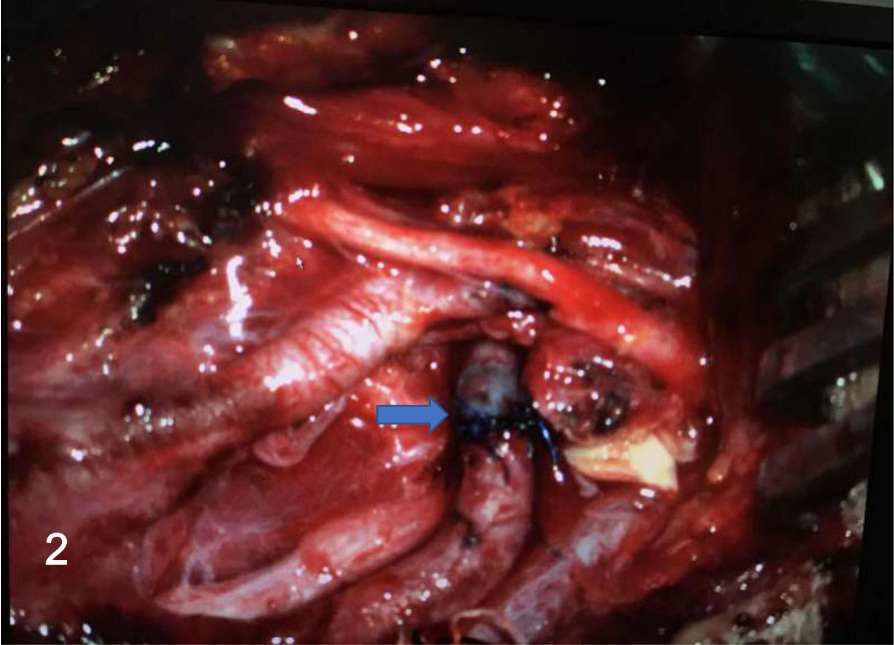
The ICA was reconstructed with end-to-end anastomosis (arrow), after the resection of the aneurysm

**Fig. 3:**
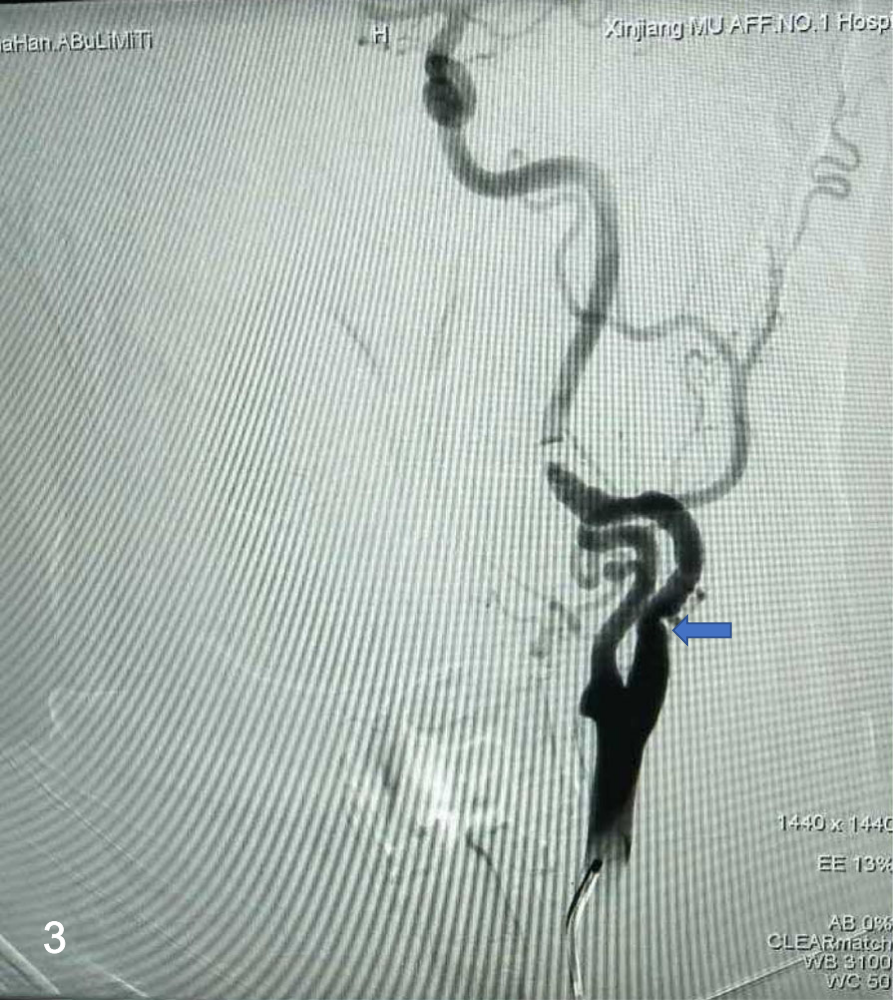
Cerebral DSA at the 5-month follow-up showed stenosis of the left ICA

We had performed the operation under general anesthesia and made an incision along the anterior sternocleidomastoid border on the left neck, exposing the carotid bifurcation, the proximal and the distal parts of the ICA carefully. Both of the proximal and distal parts of the aneurysm were clipped temporarily to complete the aneurysm resection. The continuity of the ICA was restored with end-to-end anastomosis between the two parts of the ICA (Fig. 2). Without significant abnormal findings or any complications, the patient was discharged home on the post-operative day 7. Up to the 5 months follow-up period, the patient also had slight intermittent headache and dizziness, but digital subtraction angiography (DSA) showed excellent repair of the ICA only with a slight stenosis (Fig. 3).

### Case 2

**Fig. 4:**
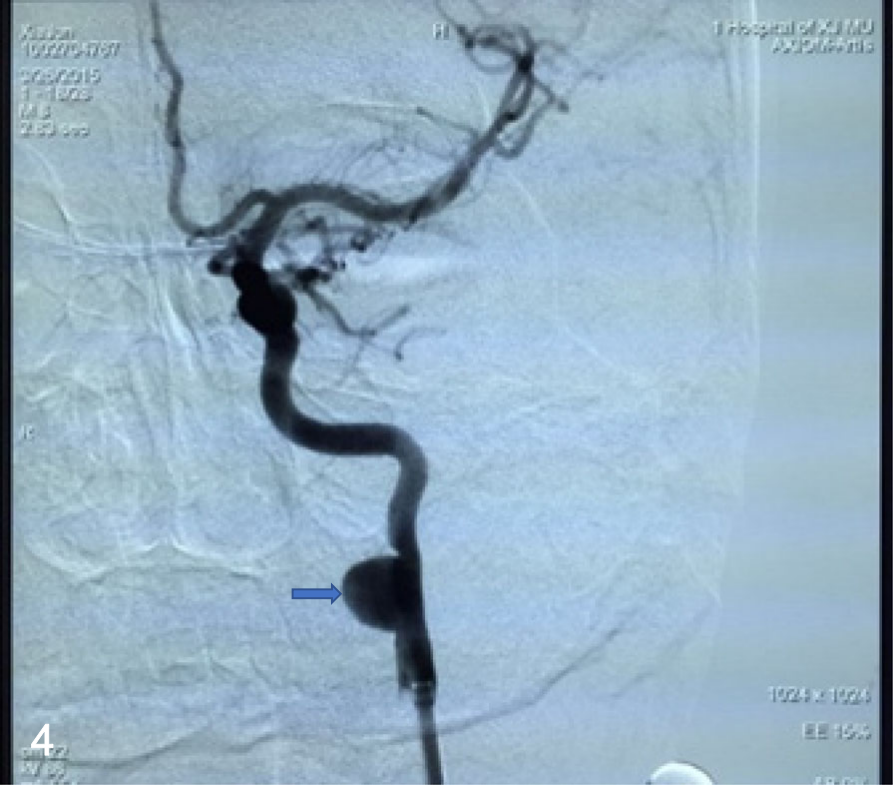
DSA showed a wide-neck EICA aneurysm with a maximal diameter of 9.8 × 10.5 mm at the C2 segment (arrow)

A 31-year-old male with a history of numbness of the right upper limb for 1 month was admitted into our department and denied the medical history of cervical infection, trauma, or operation. No abnormalities were observed on physical examination. DSA demonstrated an aneurysm at the left EICA (Fig. 4).

**Fig. 5:**
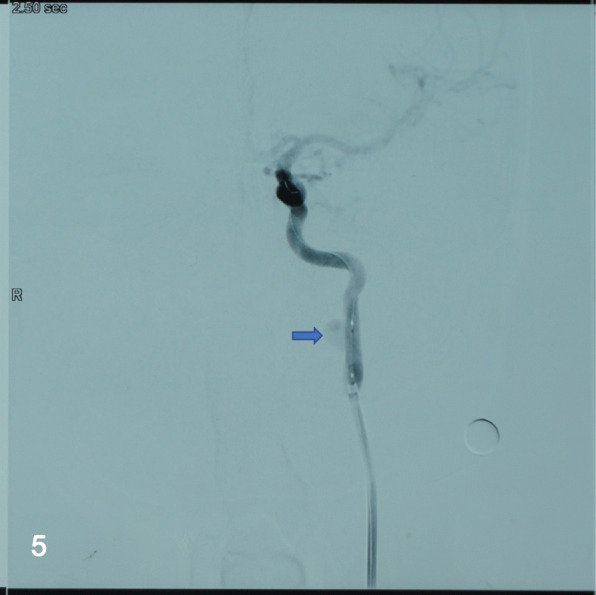
After a WCS measured4.5 mm × 16 mm was deployed, angiogram demonstrated the stent was in a good position

**Fig. 6:**
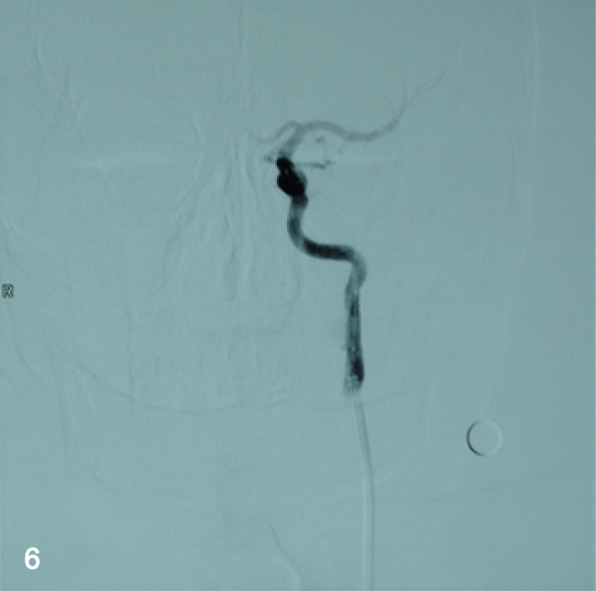
At a 7-month follow-up period, cerebral angiogram demonstrated the complete disappearance of the aneurysm

The patient took a daily 100 mg of aspirin and 75 mg of clopidogrel for consecutive 3 days before the operation. Under local anesthesia, the patient was thoroughly heparinized, then the right femoral artery was canalized with an 8 Fr. super-long sheath (Shuttle TM sheath, Cordis), and an 8 Fr. guiding catheter (Cordis, Miami, FL, USA) was positioned in the proximal segment of the left ICA, and a PTCA guidewire (Boston, USA) was navigated into the distal part of the aneurysm, subsequently, a Willis covered stent (WCS) navigated over the microwire and bridged the aneurysm neck under a roadmap guidance (Fig. 5). After the Willis has been deployed and expanded completely, an immediate final angiogram was performed (Fig. 6). The patient was discharged on the postoperative day 4 with aspirin (100 mg/day) and clopidogrel (75 mg/day). At a 7-month follow-up period, DSA demonstrated complete disappearance of the aneurysm and good patency of the EICA. Besides, the patient stayed symptom free and kept on lifelong aspirin consumption but stopped the clopidogrel.

### Case 3

**Fig. 7:**
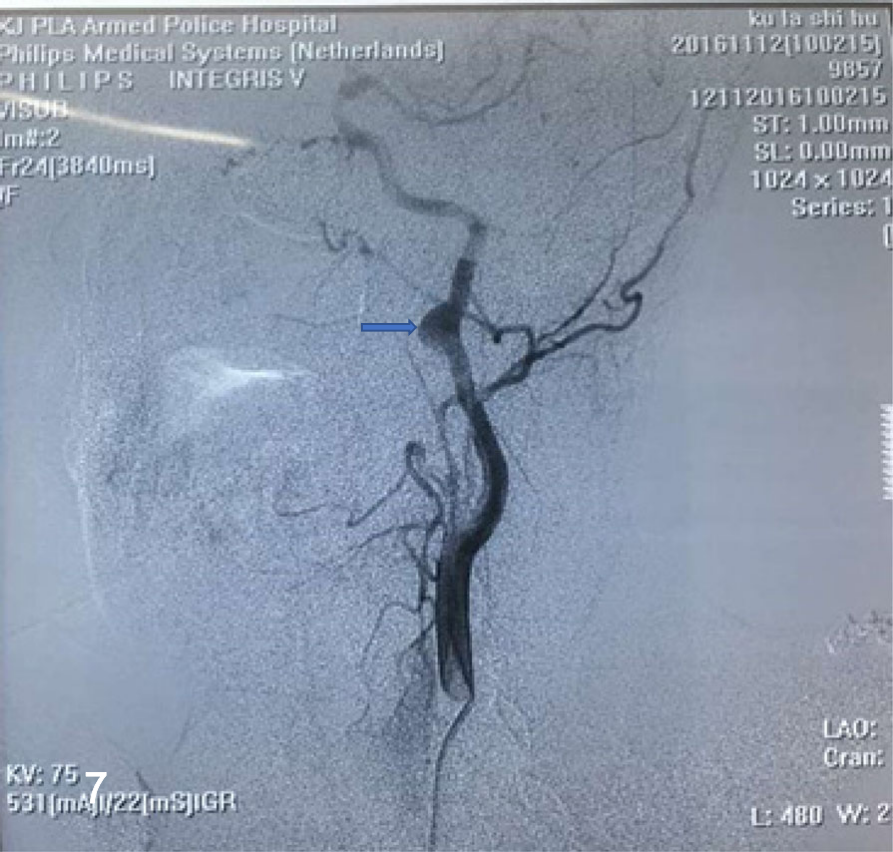
DSA revealed an aneurysm at the distal part of the straight left EICA (arrow)

**Fig. 8:**
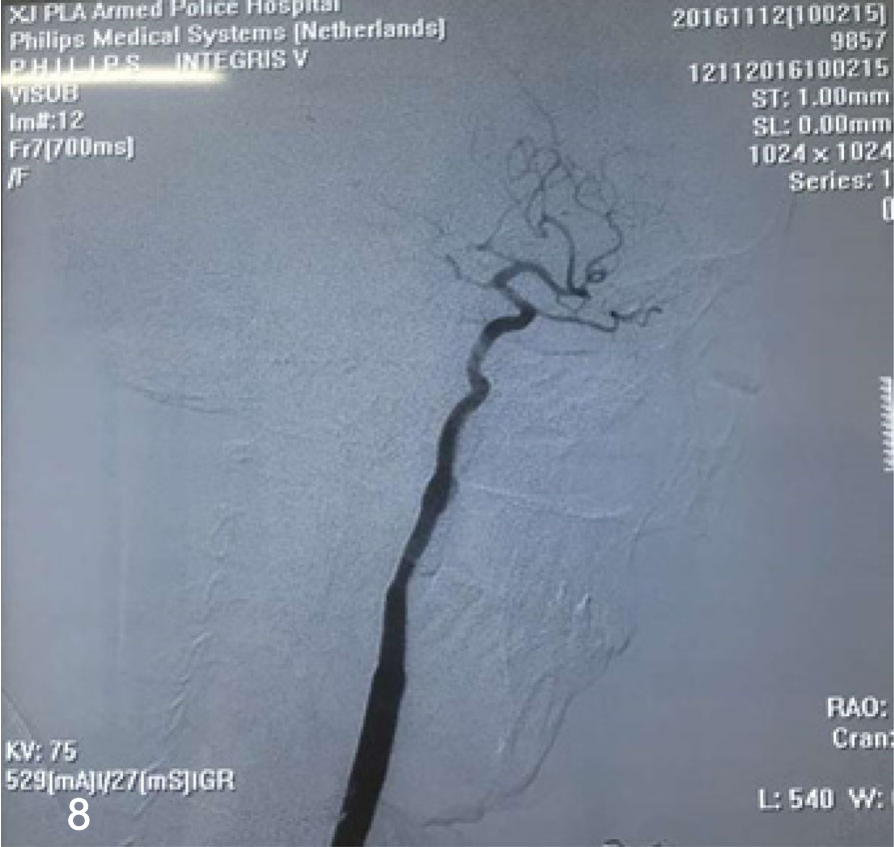
After a 4.5 mm × 16 mm WCS was implanted, angiography revealed the aneurysm disappearance

A 44-year-old female with a history of a pulsatile mass on the left side of her neck for 2 months was admitted into the neurosurgery department. She was in good condition without past medical history, only a pulsatile mass with systolic bruit was observed on physical examination. DSA (Fig. 7) confirmed an aneurysm located distally in the left EICA that was regarded responsible for the symptom. After detailed explanation and consent of the patient, we performed the endovascular treatment with a Willis covered stent. The procedure was same to the abovementioned case 2 in detail (Fig. 8). Without neurological sequels when the patient was discharged, she has been noticed to take clopidogrel for 6 months and aspirin for lifetime. At the 12 -month follow-up on telephone, the patient remained free from neurological deficits.

## Discussion

The definition of an extracranial carotid artery aneurysm (ECAA) is regarded as a dilation by 1.5 times or more of the diameter of the expected normal carotid artery [3]. The specific causes of the ECA aneurysms are still unknown, but the most common cause is atherosclerosis in old patients, besides trauma, fibromuscular dysplasia, prior surgery, congenital malformation, infection, and radiation which can all attribute to aneurysmal degeneration [4].

Aneurysms of ECA are rare manifestations and asymptomatic in most patients, but a pulsating mass or local pain in the neck [5, 6]. Patients might also experience ischemic stroke, transient ischemic attack, and amaurosis fugax because of the high possibility of natural embolization in the aneurysm or the thromboembolic complication caused by the drop-off embolus. The symptoms such as dysphasia, auricular pain, Horner syndrome, and voice hoarseness are caused by local compression of mass effect [7, 8]. And a very small number of cases present hemorrhagic complications caused by the rupture of aneurysms. Hence, it is of vital importance to recognize, diagnose, and manage it before the life-threatening complications. With the frequent use of various imaging techniques, the early diagnosis of asymptomatic aneurysms has increased compared to the earlier researches that reported nearly 50% of patients presenting with neurologic symptoms such as stroke or transient ischemic attack [5, 9, 10].

Management of EICAAs is determined by the characteristics like etiology, size, and location of the aneurysm and other relevant comorbidities in patients [5]. Although there is no consensus in ECAA management, operative therapy has been recommended in most cases especially with symptomatic or growing aneurysms to avert disastrous consequences, because of the high mortality risk in non-operated cases [11, 12]. Welleweerd et al. [11] reported that the 30-day mortality rate and stroke rate in conservatively treated patients were 4.67% and 6.67% and after surgery 1.91% and 5.16%, respectively.

As far as hemodynamics is concerned, small EICAAs are either asymptomatic or have a lower risk of ischemic stroke than large aneurysms. However, Ghilardi et al. [13] strongly advocates the need for surgical treatment of EICAAs regardless of their size, for EICAAs are reckoned as emboli source to the brain at any stage of development. In addition, the severity of brain damage and related symptoms as well as endoluminal debris generation in the operation process correlate with the size of EICAAs. Therefore, small EICAAs that are detected at an early stage should be operated promptly, because at this stage, their treatment is safer than that of large EICAAs [13]. Knowing that they had been detected aneurysms on the neck, both of the last two patients became nervous and willing to take surgical treatment eagerly in the sake of avoiding unexpected fatal complications. After a comprehensive discussion with the patients and their legal guardians regarding the risk and benefits of invasive treatment, especially the potential risk of embolization and antithrombotic agents, they chose to undergo surgery.

Operative options include endovascular and surgical approaches. Since Cooper’s first operation in 1805, open surgical intervention has been using as a first-line treatment and favored to be used when the symptoms such as mass effect or bleeding occurred. Garg et al. [14] point out the following five kinds of interventions: aneurysm clipping, excision with primary anastomosis, excision with interposition graft, extracranial-intracranial bypass, and carotid ligation. Recently, simple carotid ligation was used rarely and regarded as a last resort with a risk of 25% stroke and 20% mortality rate [15]. Reconstruction by resection of aneurysm with a direct end-to-end anastomosis is frequently used to ICA saccular aneurysms above the bulb, especially when the carotid artery is torturous. Vein or prosthetic grafts are the best alternatives when primary anastomosis is hard to perform for the fusiform aneurysm of the ICA or the CCA [9]. Aneurysmorrhaphy is used when the artery wall is intact and angioplasty is feasible but results in the secondary stenosis or occlusion of the parent artery. Extracranial-intracranial bypass may be more effective to the patients whose aneurysms extended to external skull base and when it is hard to expose distal ICA, but this method only can be used in highly selected patients with a decent donor artery. And there are two anastomoses in bypass surgery, so the occurrence of ischemic stroke caused by anastomotic stenosis or artery occlusion may be higher than other surgical approaches. The early and long-term outcome of surgical treatment in ECAA is favorable. However, cranial nerve damage after surgery occurs frequently [11]. Fankhauser et al. [4] reported that open surgical treatment was more commonly used to the patients with symptomatic and true aneurysms, while pseudoaneurysms were more likely to be treated by endovascular therapy. The treatment of ICA aneurysms is more challenging when ECAAs are distal to the common carotid bifurcation that is difficult to completely expose the lesion, an extremely tortuous common carotid artery or unfavorable aortic arc anatomy which is more complicated for endovascular options. Besides, due to limitations in the reporting of the results and confounding by indication in the available literature, it is hard to determine the optimal treatment strategy. In the first case, as the aneurysm locates relatively low at an extremely torturous artery that can be elongated, both proximal and distal portions of aneurysm are intact without any dissection. Therefore, we performed the resection of the EICA aneurysm with end-to-end anastomosis.

Endovascular treatment of ECA aneurysms has been depicted as a quick and less invasive method with the use of flow diverter devices (FDD), covered stents, bare metal stents alone, or combined with coil embolization that can lead to fewer nerve injuries and shorter convalescent time [16–18]. Endovascular treatment can also be applied to patients whom with severe comorbidity or “hostile neck” unsuitable for surgery. Covered stent as a latest developed material applied in endovascular therapy can exclude the blood flow from aneurysms immediately so that it can reduce complications associated with rupture or local compression [19, 20]. However, with its rigidity, covered stent cannot tightly attach to the vessel walls when the vessel is torturous or has a risk of occlusion of the parent artery and branches. Alaraj et al. [19, 21] reported the immediate complications resulted from using covered stents in the ECAA including arterial dissection, arterial occlusion, intracerebral hemorrhage and embolic stroke were recorded in 9.1%. Considering the superiority of long-term outcomes, stent-assisted coil embolization can be an important treatment strategy for aneurysms. Geyik et al. [22] reported that complete occlusion of the aneurysm sac with the stent-assisted coil embolization in the last follow-up data was 90.8%, while recanalization and recurrence rate at 6 months were 8%. The novel developed flow diverters (FD), such as pipeline embolization device (PED), can accelerate aneurysm occlusion with a higher occlusion rate than the usual coiling methods through a process of endoluminal reconstruction of the parent artery and by rectifying blood flow away from the aneurysm sac [23–25]; however, it takes quite a period of time to complete occlusion aneurysm sac and cannot obliterate the mass effect timely. Additionally, FD poses a high risk of progressive recanalization and delayed aneurysm rupture due to the incomplete obliteration of giant aneurysms. A multicenter study proved the safety and efficacy of flow diverters with complete occlusion rates of 71–86% and no recurrence [26, 27]. In both case 2 and case 3, the ICA of the patients was straight without excessive tortuosity or branches and the wide-necked aneurysms are located at a relatively high level; separately in C2 and C1 segments, it is difficult to completely expose the aneurysm on the neck and perform a microsurgical operation; therefore, a WCS was used to occlude the blood flow into the aneurysmal lumen which is relatively simple and fast to perform with minimal risk of procedural-related rupture of aneurysms or re-bleeding [28]. However, after the endovascular treatment, patients need to take the antithrombotic agents which consists risk of having hemorrhage. The safety and efficacy of endovascular repair on ECAA have been reported only in small case series [11, 29]. It will be necessary to summarize cases and verify long-term results.

## Conclusion

As described above, treatment methods for ECA true aneurysms are various and the choice between endovascular repair and conventional surgery depends on different factors, such as the types and locations of aneurysms, comorbidities, symptoms, and the experience and preference of the surgeon. In conclusion, we have shown that both the resection of aneurysm with an end-to-end anastomosis and utilization of WCS to ECAA are effective and safe procedures; there were neither stroke nor major neurologic complications in our cases. Controlled comparative studies are necessary to be performed in the future, but the extremely low incidence of lesions is a challenge to the design of such studies.

## Abbreviations

CTA: Computed tomography angiography
DSA: Digital subtraction angiography
ECA: External carotid artery
ECAA: External carotid artery aneurysm
EICA: External internal carotid artery
ICA: Internal carotid artery
WCS: Willis covered stent

## References

[1] Kakisis JD, Giannakopoulos TG, Moulakakis K, Liapis CD (2014). Extracranial internal carotid artery aneurysm. J Vasc Surg.

[2] Rivera-Chavarria IJ, Alvarado-Marin JC (2016). Endovascular repair for an extracranial internal carotid aneurysm with cervical access: a case report. Int J Surg Case Rep.

[3] Welleweerd JC, Nelissen BG, Koole D, de Vries JP, Moll FL, Pasterkamp G (2015). Histological analysis of extracranial carotid artery aneurysms. PLoS One.

[4] Fankhauser GT, Stone WM, Fowl RJ, O’Donnell ME, Bower TC, Meyer FB (2015). Surgical and medical management of extracranial carotid artery aneurysms. J Vasc Surg.

[5] El-Sabrout R, Cooley DA (2000). Extracranial carotid artery aneurysms: Texas heart institute experience. J Vasc Surg.

[6] Mitrev Z, Jankulovski A, Bozinovska B, Hristov N (2011). Surgical treatment of giant extracranial internal carotid artery aneurysm. J Vasc Surg.

[7] Longo GM, Kibbe MR (2005). Aneurysms of the carotid artery. Semin Vasc Surg.

[8] Yamamoto S, Akioka N, Kashiwazaki D, Koh M, Kuwayama N, Kuroda S (2017). Surgical and endovascular treatments of extracranial carotid artery aneurysms-report of six cases. J Stroke Cerebrovasc Dis.

[9] Attigah N, Kulkens S, Zausig N, Hansmann J, Ringleb P, Hakimi M (2009). Surgical therapy of extracranial carotid artery aneurysms: long-term results over a 24-year period. Eur J Vasc Endovasc Surg.

[10] Radak D, Davidovic L, Vukobratov V, Ilijevski N, Kostic D, Maksimovic Z (2007). Carotid artery aneurysms: Serbian multicentric study. Ann Vasc Surg.

[11] Welleweerd JC, den Ruijter HM, Nelissen BG, Bots ML, Kappelle LJ, Rinkel GJ (2015). Management of extracranial carotid artery aneurysm. Eur J Vasc Endovasc Surg.

[12] Welleweerd JC, Moll FL, de Borst GJ (2012). Technical options for the treatment of extracranial carotid aneurysms. Expert Rev Cardiovasc Ther.

[13] Ghilardi G, Massetto N, Cattalini C, Odero A, De Monti M, Gobatti D (2001). Brain involvement in extracranial internal carotid artery aneurysms. Vasa.

[14] Garg K, Rockman CB, Lee V, Maldonado TS, Jacobowitz GR, Adelman MA (2012). Presentation and management of carotid artery aneurysms and pseudoaneurysms. J Vasc Surg.

[15] McCann RL (1990). Basic data related to peripheral artery aneurysms. Ann Vasc Surg.

[16] Bellosta R, Sesana M, Baglini R, Luzzani L, Talarico M, Sarcina A (2008). Endovascular treatment of a symptomatic carotid artery aneurysm with a stent graft. Vasc Endovasc Surg.

[17] Huyzer M, Reijnen MM, Sybrandy JE, Buth J, Zeebregts CJ (2011). Interposition grafting of large extracranial carotid aneurysm. Tex Heart Inst J.

[18] Zhou W, Lin PH, Bush RL, Peden E, Guerrero MA, Terramani T (2006). Carotid artery aneurysm: evolution of management over two decades. J Vasc Surg.

[19] Alaraj A, Wallace A, Amin-Hanjani S, Charbel FT, Aletich V (2011). Endovascular implantation of covered stents in the extracranial carotid and vertebral arteries: case series and review of the literature. Surg Neurol Int.

[20] Welleweerd JC, de Borst GJ, de Groot D, van Herwaarden JA, Lo RT, Moll FL (2015). Bare metal stents for treatment of extracranial internal carotid artery aneurysms: long-term results. J Endovasc Ther.

[21] Chang FC, Lirng JF, Luo CB, Guo WY, Teng MM, Tai SK (2007). Carotid blowout syndrome in patients with head-and-neck cancers: reconstructive management by self-expandable stent-grafts. AJNR Am J Neuroradiol.

[22] Geyik S, Yavuz K, Yurttutan N, Saatci I, Cekirge HS (2013). Stent-assisted coiling in endovascular treatment of 500 consecutive cerebral aneurysms with long-term follow-up. AJNR Am J Neuroradiol.

[23] Chalouhi N, Tjoumakaris S, Dumont AS, Gonzalez LF, Randazzo C, Starke RM (2013). Treatment of posterior circulation aneurysms with the pipeline embolization device. Neurosurgery.

[24] Lanzino G, Crobeddu E, Cloft HJ, Hanel R, Kallmes DF (2012). Efficacy and safety of flow diversion for paraclinoid aneurysms: a matched-pair analysis compared with standard endovascular approaches. AJNR Am J Neuroradiol.

[25] Miyachi S, Hiramatsu R, Ohnishi H, Yagi R, Kuroiwa T (2017). Usefulness of the pipeline embolic device for large and Giant carotid cavernous aneurysms. Neurointervention.

[26] Becske T, Brinjikji W, Potts MB, Kallmes DF, Shapiro M, Moran CJ (2017). Long-term clinical and angiographic outcomes following pipeline embolization device treatment of complex internal carotid artery aneurysms: five-year results of the pipeline for uncoilable or failed aneurysms trial. Neurosurgery.

[27] Kallmes DF, Hanel R, Lopes D, Boccardi E, Bonafe A, Cekirge S (2015). International retrospective study of the pipeline embolization device: a multicenter aneurysm treatment study. AJNR Am J Neuroradiol.

[28] Li MH, Li YD, Gao BL, Fang C, Luo QY, Cheng YS (2007). A new covered stent designed for intracranial vasculature: application in the management of pseudoaneurysms of the cranial internal carotid artery. AJNR Am J Neuroradiol.

[29] Li Z, Chang G, Yao C, Guo L, Liu Y, Wang M (2011). Endovascular stenting of extracranial carotid artery aneurysm: a systematic review. Eur J Vasc Endovasc Surg.

